# Influence of Cementation Mode and Ferrule Design on the Fatigue Resistance of Monolithic Zirconia Endocrowns

**DOI:** 10.3390/jcm13041165

**Published:** 2024-02-19

**Authors:** Milan Stoilov, Tobias Boehmer, Lea Stoilov, Helmut Stark, Michael Marder, Norbert Enkling, Dominik Kraus

**Affiliations:** 1 Department of Prosthodontics, Preclinical Education and Dental Materials Science, Bonn University, 53111 Bonn, Germany; tobias.boehmer@ukbonn.de (T.B.); lea.stoilov@ukbonn.de (L.S.); helmut.stark@ukbonn.de (H.S.); michael.marder@ukbonn.de (M.M.); norbert.enkling@unibe.ch (N.E.); dominik.kraus@ukbonn.de (D.K.); 2 Department of Reconstructive Dentistry and Gerodontology, Bern University, 3012 Bern, Switzerland

**Keywords:** endocrown, post-endodontic restoration, CAD/CAM, fracture load, ferrule design

## Abstract

**Background**: Classic endocrowns made of dental ceramics are considered a promising alternative to traditional post-endodontic restorations. The use of circular ferrules in endocrowns is a topic of controversial discussion. Therefore, the present study aims to evaluate the effect of ferrule design and cementation mode on the fatigue resistance of zirconia endocrowns. **Methods**: Eighty human molars were divided into four groups (*n* = 20): NFC (no-ferrule, conventional cementation), NFA (no-ferrule, adhesive luting), FC (ferrule, conventional cementation) and FA (ferrule, adhesive luting). Both the classic and the modified endocrown preparation with a two-millimeter ferrule design were carried out. Endocrowns were fabricated from zirconia using the CEREC system. After thermocycling, specimens were loaded according to the step-stress test up to 1500 N. **Results**: Failure rate was low; 88.8% of total specimens passed the step-stress test. Fractures were distributed between all groups; no significant differences in fatigue resistance were detected for preparation design and cementation mode. **Conclusions**: Endocrowns appear to be a promising concept for endodontically treated molars. Ferrule and also cementation mode have only a minor influence on fatigue resistance of zirconia endocrowns. However, at very high forces, the marginal area of the ferrule represents a weak point.

## 1. Introduction

Post-endodontic reconstruction of severely damaged teeth still remains a major challenge for dental practitioners [1]. The loss of tooth structure weakens the remaining substance and increases the risk of fracture [2], potentially resulting in tooth loss [3]. Especially, teeth exposed to high bite forces require adequate restoration after endodontic treatment to minimize risk of fracture, to provide a coronal seal, and to restore functionality [4]. The most commonly used types of restoration in these cases are post and core build-ups and full crowns [3,5]. However, it is generally agreed that post preparation represents an additional iatrogenic weakening of the residual tooth structure, thus increasing the risk of fatal fracture and significantly worsening the prognosis of the tooth [6]. In addition, root canal posts may not be a viable option in certain canal configurations, such as dilated or calcified canals [7].

In this context, the endocrown concept is a minimal-invasive, defect-oriented approach to restoration that aims to better preserve the remaining tooth structure [8]. The endocrown is defined as an adhesive monolithic ceramic restoration anchored in the pulp chamber, exploiting the micromechanical retention properties of the pulp-chamber walls [8]. It does not require an adhesive build-up, thereby simplifying the entire workflow, saving time, and costs [9]. The extension into the pulp chamber provides stability and increases the adhesive surface to improve retention of the coronal part. Endocrowns offer several advantages over traditional crowns. Due to the absence of anchorage within the tooth’s root canals, additional iatrogenic weakening of the remaining tooth structure can be avoided, and complications such as perforation or root fracture might be prevented [10,11]. Because only the first 1–2 mm are used for retention, access to the root canal system is simplified for endodontic retreatment.

Yet, these advantages only come into effect if adhesive cementation can be performed [8]. Particularly, in the case of severely damaged molars, absolute isolation is difficult to achieve, even with a rubber dam. In everyday practice, inadequate adhesive cementation might often be accepted as part of a trade-off in these cases, which might result in secondary caries or loss of retention [12,13]. Thus, as long as sufficient retention and fracture resistance are ensured, conventional cementation of zirconia-based endocrowns might be an alternative approach. However, this approach provides only low micromechanical adhesion [14], as only frictional forces are generated between the corresponding surfaces and the conventional cement [15]. Modification of the preparation design by applying a circular shoulder thus appears to be a reasonable solution, as it might provide additional friction, sufficient cement flow [16], and a proper marginal seal [16].

It is known that endodontically treated teeth are more prone to fractures than vital teeth [17]. Fracture resistance depends on various factors, including the quality and quantity of remaining tissue, loading conditions, and restoration type [3,18,19]. The presence of a ferrule as a reinforcing or stress-reducing element is therefore widely debated [20,21]. The functional principle of the ferrule relies on its protective role in the cervical part of the prosthetic crown, surrounding the marginal remaining tooth structure and acting like a “ring” to prevent bending and strengthen the functional unit of tooth and restoration. Additionally, it enhances contact between the prosthetic crown and the remaining root, improving retention. According to classical prosthetic principles, the ferrule should encircle the sound tooth structure and be at least 1 to 2 mm high, with parallel axial walls. Numerous studies confirm the positive effect of a ferrule in post-endodontic reconstruction; however, most of them are focused on conventional crown designs [22,23,24,25,26,27].

Still, the literature on ferrule design on endocrowns is partly contradictory. The systematic review conducted by Mostafavi et al. [28] revealed a strong heterogeneity in the examined preparation designs, which complicated a definitive assessment. Although some authors claim that ferrule-containing endocrowns exhibit significantly higher fracture loads [29,30], others show that there is no difference in fracture resistance or mode of failure [31]. Although standard endocrown preparations include a 90° butt joint to increase strength against compressive forces, a shoulder preparation is recommended at the same time, as this would create an additional axial wall [32]. The shoulder is further thought to increase fracture strength values and provide improved force distribution and a reduced thickness of the luting cement, resulting in lower shrinkage polymerization [30]. Mostafavi et al. [28], nevertheless, recommend adherence to a butt joint design, as they consider the preservation of residual tooth structure to be the highest priority. In addition, a butt joint design is recommended as a simple and efficient margin design for the practitioner.

The beneficial impact of ferrule design in indirect adhesive restorations like endocrowns remains to be proven, as only a limited number of in vitro studies have been conducted on this subject. Einhorn et al. [29] demonstrated that the inclusion of an additional ferrule in the preparation design of lithium disilicate molar endocrowns improved their fracture resistance, although no significant difference in load-bearing capacity was observed between preparations with a 1 mm or 2 mm high ferrule. Similarly, Taha et al. [30] reported comparable findings for polymer-infiltrated ceramic endocrowns. A finite element study by Alberto et al. [33] showed that the use of a 2 mm high ferrule design would significantly reduce stress peaks in the tooth root. They even recommend a partial ferrule if a full ferrule is not feasible, rather than incorporating no ferrule at all. However, a contrasting study found that classic endocrowns (without a ferrule) made of lithium disilicate-reinforced ceramic performed equally well in terms of marginal integrity and fatigue resistance when compared to standard post-and-core crown restorations (with ferrule) [34].

To the best of authors’ knowledge, the influence of ferrule design and simultaneous conventional cementation on fatigue resistance of zirconia endocrowns has not yet been investigated. In the majority of investigations assessing the fracture resistance of endocrowns, specimens are subjected to a standardized static testing protocol that involves the application of compressive loads and utilizes a load-to-fracture design [35,36]. These investigations frequently expose significantly elevated stress values, substantiating the clinical applicability of endocrowns. Nevertheless, ceramics exhibit linear-elastic and brittle characteristics, rendering them susceptible to pre-existing flaws [37]. Consequently, fatigue emerges as the principal mechanism for the clinical failure of ceramics [38], given the anticipation of gradual crack propagation in oral environments [39]. Consequently, for the assessment of prolonged functional reliability, the utilization of staircase or step-stress protocols is recommended [40].

Therefore, the purpose of this study was to determine the effect of ferrule features and shoulder finish line on endocrown restoration fatigue strength. The first null hypothesis was that there is no difference in fatigue resistance between conventional endocrown restorations and endocrowns with modified preparation design. The second null hypothesis assumes no difference in fatigue resistance between conventional cementation and adhesive luting of endocrowns with and without ferrule design.

## 2. Materials and Methods

**Figure 1:**
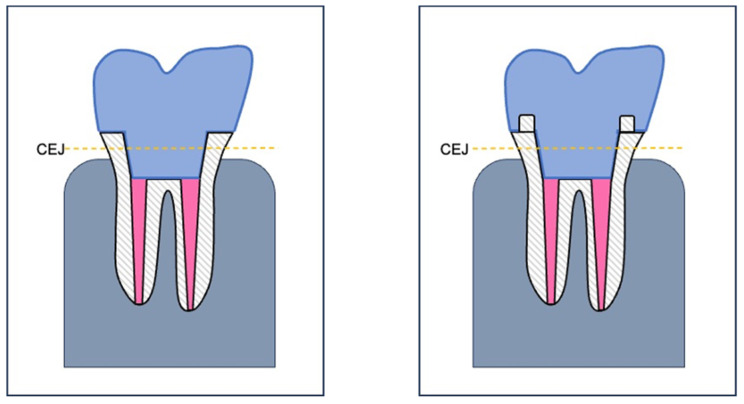
Schematic illustration of the embedded molars with endocrowns (light blue = endocrown, pink = root canal filling, white shaded = tooth root, gray blue = plastic block). These were decapitated and prepared above the cemento-enamel junction (CEJ). The (**left image**) depicts the classic endocrown (NFA, NFC), and the (**right image**) illustrates the modified endocrown design with a 2 mm ferrule and a shoulder finish line (FA, FC).

Eighty human mandibular and maxillary molars were used in this study. The extraction of teeth was conducted in accordance with standard clinical protocols, and the specimens were acquired from nearby oral and maxillofacial surgery clinics following the acquisition of informed written consent from the respective patients. To prevent dehydration and biofilm formation, teeth were stored in a NaCl solution containing sodium azide (0.9% NaCl + 0.001% NaN_3_) at a temperature of 6 °C. Cleaning of teeth was performed using airscaler (SONICflex^®^, Dentsply Sirona, Bensheim, Germany) and hand instruments (universal curettes and hand scalers). The specimens were then embedded in cylindrical acrylic blocks made of autopolymerizing denture base resin (PalaXpress^®^ clear, Heraeus Kulzer, Hanau, Germany) up to approximately 2 mm beneath the cemento enamel junction (Figure 1). The cylindrical shape of the specimen matches the mount of the fatigue testing machine, ensuring a precise fit and uncomplicated handling during the loading tests.

Specimens were then randomly divided into four groups: NFA (*n* = 20; no-ferrule, adhesive cementation), NFC (*n* = 20; no-ferrule, conventional cementation), FA (*n* = 20; with ferrule, adhesive cementation), and FC (*n* = 20; with ferrule, conventional cementation).

**Figure 2:**
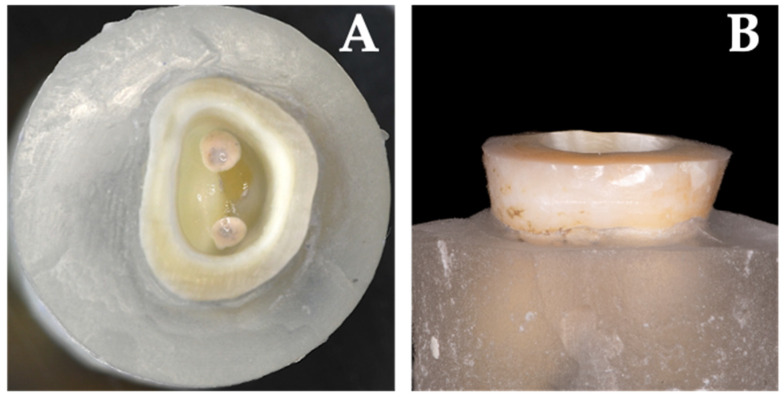
Occlusal (**A**) and lateral (**B**) view of a molar after classical endocrown preparation.

**Figure 3:**
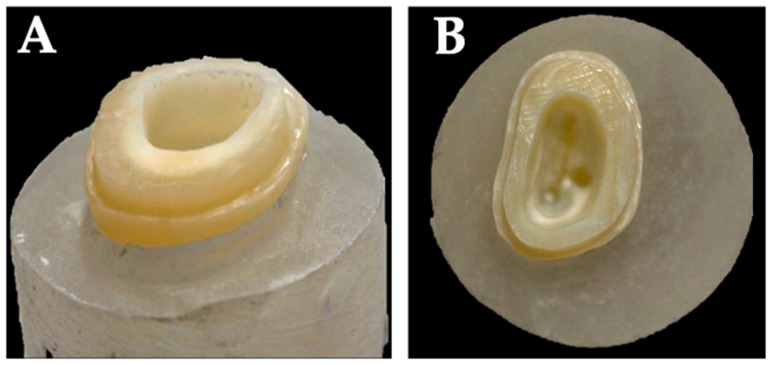
Occlusal (**A**) and lateral (**B**) view of a molar with modified endocrown preparation. A 2 mm wide shoulder finish line with the respective ferrule design is clearly visible.

To standardize preparations and to keep variance low, the same researcher completed all preparations. Initially, specimens were decapitated and finished 2 mm above the cemento enamel junction (Figure 2 and Figure 3) using a water-cooled high-speed handpiece (Expertmatic Lux E25L, KaVo©, Biberach, Germany) and diamond burs (FG 158012 and FG 158C012, Horico^®^, Berlin, Germany). Subsequently, the pulp chamber was accessed and provisional root canal treatment was performed. Canal orifices were explored using ISO 10 and 15 K-files (VDW Dental, Munich, Germany) and prepared with Gates Glidden rotary instruments (size 004 and 006, Anteos^®^ VDW Dental, Munich, Germany) to further simulate endodontic preparation of the pulp chamber and canals. Partially existing pulpal remnants were extirpated and the root canals were sealed with zinc oxide eugenol (Speiko^®^, Bielefeld, Germany) and phosphate cement (Hoffman Dental©, Berlin, Germany) up to approximately 2 mm apically to the orifices. The use of zinc oxide eugenol cement ensured a bacteria-proof seal of the root canal. However, to prevent the eugenol from impairing the adhesive bond [41,42], this layer was covered with zinc oxide phosphate cement. After the setting of the cement, existing irregularities of the pulp chamber walls were leveled and a slight divergence was prepared. Furthermore, excess was removed and transitions of the pulp walls to the occlusal area were finished with a diamond bur (FG291 C 014 Horico^®^, Berlin, Germany).

Specimens were prepared according to endocrown preparation rules in two distinct groups: NFA and NFC. This preparation entailed a 90° butt margin, smooth internal transitions, internal taper of the pulpal chamber, flat pulpal floor with sealed radicular spaces, and supragingival enamel margins if possible (groups NFA and NFC) (Figure 1 and Figure 2) [29,43]. In groups FC and FA however, the modified preparation design with an additional ferrule design and 2 mm shoulder finish line was implemented (Figure 1 and Figure 3).

**Figure 4:**
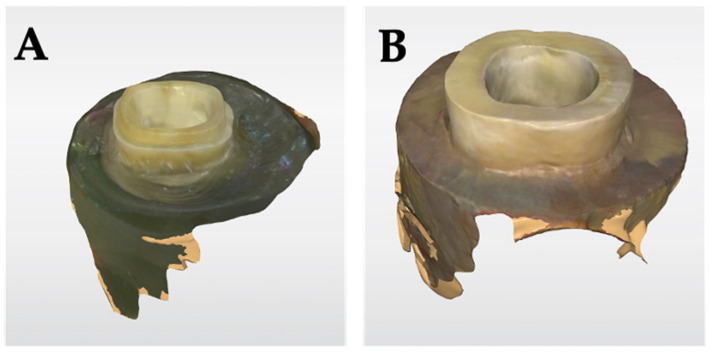
Screenshots of the scanned preparations. Scanning was performed using the CEREC^®^-Primescan ((**A**) = endocrown preparation with ferrule; (**B**) = classical endocrown preparation).

All specimens were scanned and designed using the CEREC^®^-Primescan and Software (Model-No.: D3639, Serial-No.: 102884, version: 5.2.4.276189, Dentsply Sirona©, Bensheim, Germany) and restored with a monolithic zirconia endocrown milled out of IPS e.max^®^ ZirCAD (Block B45, Ivoclar Vivadent, Schaan, Liechtenstein) (Figure 4). A spacer value of 50 μm was set for the conventionally cemented endocrowns and a value of 80 μm was set for the adhesively bonded endocrowns. The subsequent processing of the 80 restorations was carried out using CEREC^®^ MC XL (Model-No.: D3439, Serial-No.: 314461; Dentsply Sirona©‚ Bensheim, Germany). Restorations were then dried and sintered using the CEREC^®^-SpeedFire furnace (Model-No.: D3639, Serial-No.: 504609, Dentsply Sirona©, Bensheim, Germany) in accordance with the manufacturer’s recommendations. Based on the batch number, a sintering shrinkage of 20–25% was precisely implemented by the system to ensure the proper fit of the restorations. Proper seating was then verified using a disclosing media (Xantopren^®^ L, Kulzer©, Hanau, Germany) followed by thorough steam cleaning and drying with oil-free compressed air. Interfering contacts were marked and then removed with a diamond bur (FG 158 C 012 Horico^®^, Berlin, Germany) under constant water cooling.

Depending on group classification, restorations were cemented conventionally (NFC and FC) with glass-ionomer cement (Ketac™ cem plus automix, 3M Espe, Landsberg am Lech, Germany) and adhesively (NFA and FA) with Variolink esthetic^®^ DC (Ivoclar Vivadent©, Schaan, Liechtenstein). The intaglio surfaces of the restorations underwent sandblasting using aluminum oxide (Al_2_O_3_) particles (50 μm at 0.05 MPa) to generate mechanical microretention. Subsequently, they were thoroughly cleaned and degreased using ethanol. For conventional cementation, glass-ionomer cement was prepared according to the manufacturer’s instructions using an automix cartridge. The intaglio surfaces were coated with a thin layer of cement, and the endocrowns were then seated into their final position under a 100 g load [44]. After a twenty-minute primary setting time, any excess cement was removed using hand instruments.

To facilitate adhesive luting, a slender layer of a silane agent (Monobond Plus^®^; Ivoclar Vivadent©, Schaan, Liechtenstein) was meticulously applied to the intaglio surface using a microbrush, subjected to two intervals lasting 60 s each, and any surplus material was subsequently dispersed using compressed air. Tooth surfaces were prepared for luting with 37% orthophosphoric acid (Vocoid Gel^®^, Voco Dental©, Cuxhaven, Germany). Enamel surfaces were exposed to the etching gel for 60 s and dentin for 15 s followed by water rinse (30 s) and air drying (15 s). Afterwards, the Syntac^®^ Classic (Ivoclar Vivadent©, Schaan, Liechtenstein) adhesive system was used. The Syntac^®^ primer and adhesive were sequentially applied for 15 s each, followed by a meticulous removal of excess through air blowing. In the final step, Heliobond^®^ (Ivoclar Vivadent©, Schaan, Liechtenstein) was applied thinly and was light-cured for 30 s (Bluephase^®^ PowerCure, *λ* = 385–515 nm, Ivoclar Vivadent©, Schaan, Liechtenstein). Endocrowns were luted with Variolink esthetic^®^ DC (Ivoclar Vivadent©, Schaan, Liechtenstein) with a load of 100 g [24] and a two-second tack cure was applied to all surfaces, after which excess cement was removed. All surfaces received a final 40 s light cure (Bluephase^®^ PowerCure, *λ* = 385–515 nm, Ivoclar Vivadent©, Schaan, Liechtenstein) and were again stored in the above-mentioned sodium azide solution under dark conditions at 6 °C.

Prior to mechanical loading, the samples were subjected to artificial aging (thermocycling). Each sample was exposed to 10,000 cycles in water baths at 5 °C and 55 °C. Per basin, the immersion time was 60 s and the transfer time from basin 1 to basin 2 was set at 3 s.

In this investigation, the step-stress methodology was employed, incorporating cumulative damage considerations and assessing survival probability with respect to elevated load/stress and cycle quantities at each successive step. This was achieved using Kaplan–Meier survival analysis [45]. Furthermore, this approach incorporates run-outs in the analysis, utilizes varying stress amplitudes, and offers estimations for longer lifetimes [46].

**Figure 5:**
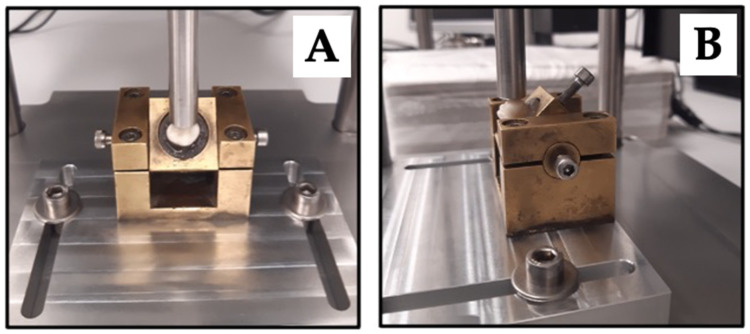
The image displays a test sample secured within a fixture, which is loaded with an 8 mm diameter cylindrical, stainless-steel piston ((**A**) = frontal view). The piston has been mounted onto the mesio-palatal cusp for the upper molars and the centro-buccal cusp for the lower molars ((**B**) = lateral view).

The fatigue resistance was tested using the CeraTest 2K testing machine (SD-Mechatronik, Feldkirchen-Westham, Germany), which was equipped with a load cell with a range from 0 to 1600 N. The chewing cycle was simulated by an isometric contraction. Specimens were placed into a fixture with the long axis of the tooth oriented at a 45° angle to the testing device (Figure 5). The palatal cusps were loaded with an 8 mm diameter cylindrical, stainless-steel piston with a unidirectional axial force and a frequency of 2.3 Hz. Each specimen was subjected to 15 steps of 10,000 cycles. The applied loading force was incrementally raised in increments of 100 N per step, reaching a maximum of 1500 N, over the course of 150,000 cycles. Specimens were loaded until fracture or to a maximum of 150,000 cycles and the number of endured cycles was registered. Continuous measurement of the counterforce exerted by the specimen allowed for the detection of premature failure by detecting force drop off. Load and number of cycles until fracture were documented for each sample and exported for statistical analysis, focusing on severe fracture as the primary event. Loads were investigated up to 1500 N, because higher forces would by far exceed the physiological maximum under normal conditions. Even in pathological conditions, no bite forces above 1500 N have been found in the literature [47,48].

In case of failure, the specimens were visually examined in order to assess which fragments were suitable for fractographic analysis. Analysis was performed visually at 25× magnification (Leica Stereo Wild + KL 2500 LCD, Leica Camera, Wetzlar, Germany). Failed specimens were analyzed for failure mode to determine whether it was a cohesive failure within the zirconia or tooth, combined failures or retention failure (adhesive failure) between restoration and tooth structure without fractures in any part. Failures involving the tooth structure were classified as restorable or non-restorable, which in a clinical situation would require tooth extraction. Classification was based on an agreement between two calibrated examiners.

Descriptive analysis was performed using “Excel” (version number: 16.78.3 (23102801)) (Microsoft Corporation©, Redmond, WA, USA) and statistical analysis using “Prism 10™”(version number: 10.1.1 (270)) (GraphPad Software©, Inc., San Diego, CA, USA). Survival analysis was conducted using the Kaplan–Meier method followed by the Mantel–Cox/log-rank test at a confidence interval of 95% (*p* < 0.05) for comparison of survival time between the groups.

## 3. Results

**Figure 6:**
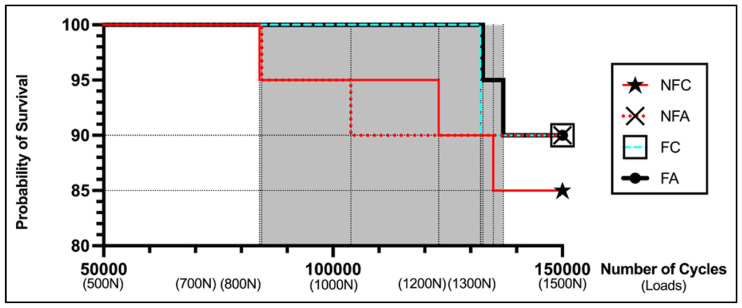
Kaplan–Meier plotted survival curves of the experimental groups. The X-axis depicts cycles and their corresponding applied forces. The gray portion indicates the area in which fractures have occurred.

After thermocycling, 71 (88.75%) of 80 specimens survived the cyclic loading up to 150,000 cycles and a final load of 1500 N. Premature failure was detected in nine specimens. All specimens survived loading up to the 80,000th cycle and up to a force of 500 N. First failures were observed at 83,950 cycles and at 600 N (Figure 6 and Table 1). Five failures occurred in the groups without ferrule design (NFC, NFA) and four failures in the groups with ferrule design and shoulder finish line (FC, FA). Furthermore, an approximately uniform distribution of failures was observed with respect to the cementation mode. Five failures were noted in the groups with conventional cementation (NFC, FC) and four failures in the groups with adhesive cementation (NFA, FA) (Table 2). Differences in survival between the groups were not statistically significant (*p* > 0.05) (Table 3).

**Figure 7:**
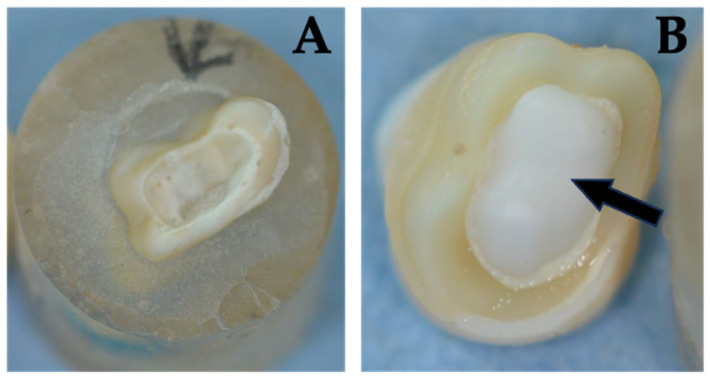
Visible is an irreparably fractured sample from the FC group. The fracture runs across the tooth root (**A**). It should be noted that the endocrown is still intact. The intrapulpal part of the endocrown is well visible (arrow) (**B**).

**Figure 8:**
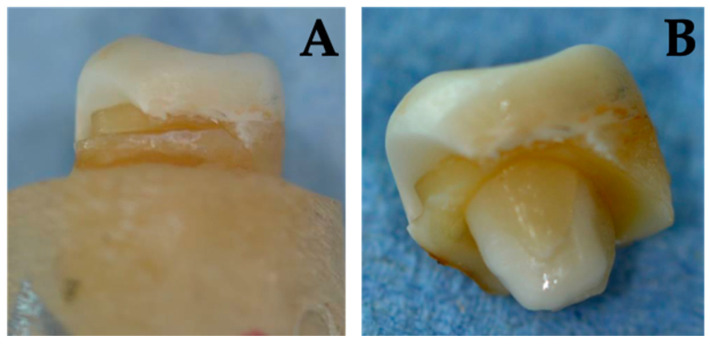
Irreparably fractured sample from the FA group. The longitudinal fracture and the involvement of the endocrown are clearly visible (**B**). However, the endocrown is exclusively fractured in the marginal area (**A**).

Three restorations experienced non-reparable fractures. However, different fracture modes were observed (Table 2): all fractured specimens of Group NFC represented reparable failures. The failures were limited to vertical infractions of the tooth structure and were located in the marginal area. In Groups NFA, FC, and FA both, restorable and catastrophic failures were observed. Restorable failures were cohesive tooth fractures that allowed preservation and crowning. Fatal fractures showed combined longitudinal fractures (tooth and endocrown) that extended deep to apical and thus could not be preserved (Figure 7 and Figure 8). Fractographic analysis revealed that failures always occurred in the direction of the loading vector and originated from the occlusal loading point and spread transversely from coronal to apical (Figure 7 and Figure 8).

## 4. Discussion

It is well known that after endodontic therapy, definitive crowning is essential to protect the remaining tooth structure from mechanical forces. Because of exposure to oral fluids, coronal sealing is also crucial as a protection against microbial recontamination of the root canal system [49]. It has been shown that delayed crowning of endodontically treated teeth is associated with a 65% risk of tooth loss after 3 years [4] and it is generally agreed that the use of conventional posts to retain the coronal core prior to crowning is often associated with an increased risk of root fracture [1]. Mismatch in the elastic modulus between post and tooth, excessive dentin removal and inadequate ferrule design are discussed as potential causative issues. Complex canal morphologies and obliterated or dilated root canals often impede the insertion of a post [4]. Particularly for the molar region, endocrowns have been established as an alternative and low-risk treatment option after endodontic therapy [43,50,51].

In the present study, intact maxillary and mandibular molars were selected for post-endodontic treatment as endocrowns are primarily recommended for molar restorations [51]. Previous research by Bindl et al. [51] and Ahmed et al. [52] showed that premolars have inadequate residual tooth structure, and the classic procedure with a post and core build-up is more suitable for premolar restorations. However, a recent meta-analysis by Thomas et al. [53] found no significant difference in fracture resistance between molars and premolars restored with endocrowns, indicating that premolars could also be potential candidates for endocrown restorations. However, the meta-analysis describes methodological limitations in the studies analyzed, indicating that further investigations are required to address the issue of restoring premolars using endocrowns. To simulate the clinical scenario of a severely damaged and non-vital tooth, the molars were decapitated and prepared at the cemento-enamel junction (CEJ). In this situation, the adhesion of the endocrown is limited to the cervical dentin, the first 2 mm of the root canal orifices, and the surfaces of the pulp chamber. Typically, under these conditions, a post and core build-up would be necessary, which is considered the traditional approach for a post-endodontic crowning.

In this study, the impact of ferrule features and cementation mode on endocrown fatigue resistance was evaluated using the step-stress approach. To the best of the authors’ knowledge, this is the first investigation employing this method for fatigue testing of zirconia endocrowns. The predominant approach involved employing compressive or static loading methods, where specimens are statically loaded until fracture. This method generates high fracture resistance loads, thereby resulting in favorable assessments of the respective material or restoration type [35]. Nevertheless, it does not accurately replicate the oral fatigue behavior of dental ceramics [46]. In order to address this aspect, dynamic loading protocols have been formulated to replicate in vivo fatigue progression over time within in vitro environments [54]. Currently, there are no existing standards for dynamic testing of ceramic restorations [55]. Therefore, test protocols have been developed to perform chewing simulations under constant physiological mechanical load, additionally including thermocycling. If no fracture occurs, a subsequent static fracture test is carried out [56,57]. In this context, chewing simulation represents an artificial aging rather than simulating fatigue over time [55]. According to Kelly et al. [22,46], the accumulation of damage over multiple cycles at lower loads can influence the durability of ceramic restorations and reduce their service life. To examine reliability over time in function more effectively, earlier studies employed staircase or step-stress methods [36,58]. Regarding this matter, the step-stress test represents an efficient fatigue testing approach, providing survival probability data at each load step [21,45]. According to Venturini et al. [37,59], the step-stress method considers slow crack growth better than the staircase test method and additionally provides information about data dispersion.

The results in this study indicate that the ferrule design does not have a statistically significant impact on the fatigue resistance of monolithic zirconia endocrowns in our setting (Table 3). A total of nine failures (11%) were observed in this study, with a nearly equal distribution between endocrowns with (four fractures) and without a ferrule design (five fractures). However, early fractures occurred more frequently in groups without ferrule design than in the other groups. Values between 600 and 900 N were observed, whereas endocrowns with ferrule design only fractured at 1400 N. All remaining samples were able to withstand the testing up to 1500 N and 150,000 cycles. Initial fractures were observed at 600 N and approximately 84,000 cycles (Table 1). Nevertheless, even these are very high forces that are rarely present in the oral cavity [60], and they could also be detrimental for other restoration types and materials. These values are especially expected during accidental biting or traumatic insults [61,62].

Considering the high survival rates of the examined specimens, it is emphasized that the statistical validity is restricted due to limited power to detect differences between the groups. The low number of events (11%) represents a limitation of our study, as the power of survival analyses depends on the number of events and not on the group size or the number of samples per se. A temporal extension of the test cycles is impractical and not realistic because the applied force is already higher than the in vivo norm. Given the survival probabilities and low failure rates, no significant differences between the groups would be expected in vivo. This is at least true within the framework of the step-stress model, which is considered convincing in this context.

Of the total nine fractured samples, only three teeth were no longer restorable and would have required extraction in a clinical setting. They exhibited catastrophic fractures with destruction of the restoration and the tooth root extending into the apical third. One catastrophic fracture was observed in a group without ferrule (FC, 700 N), and another in each of the groups with ferrule preparation (FC, 1400 N; FA, 1400 N). More catastrophic fractures might have been expected in the conventional endocrown groups, as the stabilizing effect of the ferrule was absent, potentially leading to less favorable force distribution. However, the preparation of the ferrule involves additional substance removal, weakening the tooth and possibly making it more prone to fractures. This approach theoretically contradicts the minimally invasive and defect-oriented concept of the endocrown, which aims to preserve as much tooth structure as possible through adhesive bonding. Nevertheless, it is noticeable that, in the few fractures that were observed, specimens without a ferrule design exhibited fractures at markedly lower forces compared to the groups with ferrule design. In these groups, fractures occurred only at 1400 N, which represent extremely high values. Catastrophic failures seem probable in these high force ranges, possibly not directly linked to the preparation design or substance removal but rather caused by a form of overload.

It is worth considering that, as mentioned earlier, the forces applied in the present setting far exceed those encountered in the oral cavity. On one hand, these high values demonstrate the strength of the restorations under investigation. On the other hand, it would be valuable for future research to subject the specimens to significantly more cycles at more realistic chewing forces ranging between 50 N and 300 N. Additionally, simulating the effects of biological aging in in vitro studies, even with prior thermocycling, presents challenges. Therefore, it would be advisable to pursue an in vivo model, potentially employing a split-mouth design to assess the survival rate of endocrowns and their marginal integrity compared to traditional crowns with post-core build-up. The present study was conducted under ideal conditions and in strict adherence to all manufacturer’s procedures and laboratory protocols. However, it is important to acknowledge that application errors in adhesive luting are not uncommon and can compromise bond integrity, leading to restoration failure. Furthermore, to ensure optimal standardization, healthy extracted molars devoid of caries or extensive restorations were utilized. These teeth were meticulously prepared by the researchers under magnification following root canal treatment, ensuring a minimally invasive approach and maximal preservation of the remaining tooth structure. It is worth noting that such ideal conditions may not always be achievable in clinical practice, potentially resulting in variations such as inadequate wall thickness, which could increase the risk of catastrophic fracture.

Previous studies on the fracture resistance of traditional endocrowns have demonstrated their ability to withstand fracture loads ranging from 674 N to 2606 N [36,63,64]. Lin et al. [65] as well as Dejak and Mlotkowski [50] also confirm in their finite element analyses that traditional endocrown restorations exhibit lower internal stress forces compared to the conventional approach with a full crown and post-core build-up. However, a recent finite element analysis by AboElhassan et al. [66] demonstrates that endocrowns with a ferrule design exhibit better stress distribution and magnitude compared to traditional endocrowns. Einhorn et al. [29] also show that endocrowns with integrated ferrule design could withstand significantly higher loading forces than traditional endocrowns. In addition to this, they showed that the height of the Ferrule design is crucial, as they observed less catastrophic failure with a 1 mm height compared to a 2 mm height. Furthermore, it was observed that the marginal area of the endocrowns with ferrule design was consistently involved in the fracture of the samples. This, in contrast to the classic design, appears to be a potential weak point of the ferrule. It is presumed that in certain areas, possibly due to the minimally invasive approach, the adequate material layer thickness was not maintained. Therefore, the authors recommend always ensuring at least a 2 mm shoulder finish line to maintain sufficient thickness of the crown margin when employing endocrowns with ferrule design.

Similar to the conventional approach for post-endodontic treatment, smaller diameter posts are recommended to preserve more tooth structure and thereby enhance the fracture resistance of post-restored teeth [19]. In this context, the ability to withstand fractures appears to be directly and positively correlated with the amount of surrounding dentin wall thickness around the post. A study by Farina et al. [67] demonstrated that fracture resistance at 1 mm or 2 mm of remaining dentin thickness was significantly higher than at 0.5 mm. This might support our observations, as most fractures observed in our study represented cohesive fractures in the root. The remaining fractures were combined fractures, cohesive both in the tooth and in the restoration. Inadequate remaining dentin thickness might have been a primary factor leading to tooth failure and subsequently, restoration failure. Two-thirds of the failures could have been restored in a clinical setting. This can be attributed to the moderate substance removal during the minimally invasive approach in endocrown preparation and the absence of post drilling, which helped to preserve tooth structure. Therefore, remaining dentin thickness is a crucial factor for the survival of post-endodontically treated teeth (especially for the tooth itself after endocrown failure). The decision whether to use an endocrown with or without ferrule design should be made considering the clinical situation. In cases of limited remaining dentin thickness, standard ferrule preparation should be avoided to prevent further weakening of the tooth. Conversely, in the presence of an adequate amount of circular tooth structure, employing a ferrule design would be advisable based on the current body of research. In accordance with Einhorn et al. [29] a ferrule design with a height of 1 mm should be accomplished. However, in cases of excessive weakening of the tooth, especially with defects below the cemento-enamel junction, the use of a conventional crown with post-core build-up is recommended [66].

The success of endocrown restorations is notably influenced by the manner in which the restoration adheres to the tooth, underscoring the crucial role of achieving optimal bonding for augmenting their mechanical efficacy and ensuring long-term durability under oral functional conditions [68]. According to a systematic review by Papia et al. [19], fractures and retention loss represent the most common complications for endocrowns. However, the present study exclusively focused on the fatigue resistance of conventionally and adhesively cemented endocrowns with and without ferrule.

No significant difference in fracture resistance was observed between conventionally cemented (glass-ionomer cement) and adhesively bonded endocrowns. Therefore, cementation mode (adhesive versus conventional) appears to have no influence on the fracture rate of zirconia endocrowns with and without ferrule. In the present study, glass-ionomer cement (GIC) was used as the conventional cement, which was based on its cost-effectiveness and non-technical sensitivity [69]. GICs exhibit good biocompatibility [70] and cover a wide range of clinical indications. In contrast to resin luting cements, absolute isolation is not necessary when using glass-ionomer cement for cementation [71], making it a suitable alternative cement for subgingival defects under relatively dry conditions. The use of an automix cartridge in the current study, without requiring additional conditioning of the restoration or the tooth, helped to avoid technique-related errors during cementation. This is particularly advantageous in a clinical setting and additionally saves time. Fracture rates of zirconia endocrowns cemented with glass-ionomer cement have not been investigated thus far. Due to the almost exclusive use of ceramic materials (resin ceramic, feldspathic, and lithium disilicate) [72,73], primarily adhesive luting cements are logically used to achieve adequate bonding between the restoration and the tooth structure. This bonding is crucial in supporting the ceramic material against fractures. Adhesive systems, known for their technique sensitivity, have proven effective in enhancing the fracture strength of the ceramic material [74]. Regarding endocrowns, it has also been demonstrated that adhesive bonding can be successfully applied, as its utilization correlates with good survival rates of the restorations [75]. Our findings indicate that concerning fracture resistance, endocrowns cemented with glass-ionomer exhibited comparable performance to those retained adhesively. Therefore, conventional cementation seems to offer a promising alternative to adhesive composite cements, particularly in scenarios where achieving complete dryness is challenging.

## 5. Conclusions

Given the limitations of this study, the application of zirconia-based endocrowns emerges as a promising alternative to the conventional approach, which involves post-core build-up followed by subsequent crowning in dental procedures. In vitro assessments of the endocrowns in this study demonstrate promising fatigue resistance using the step-stress approach. The presence of a circular ferrule with a 2 mm wide shoulder did not significantly impact fatigue resistance. However, further in vivo studies are essential to evaluate their performance in clinical settings, considering realistic biological aging.

Additionally, no significant difference in fatigue resistance was observed between conventionally cemented endocrowns and those adhesively bonded. Therefore, conventional cementation might offer a less technique-sensitive and reliable luting method for endocrowns. Nevertheless, further studies should investigate the retention forces of endocrowns conventionally cemented with glass-ionomer cement compared to adhesively cemented endocrowns. Marginal integrity or infiltration should also be investigated in this context.

## Figures and Tables

**Table 1:** The number of failures and their percentage distribution are presented. Additionally, the forces and cycle numbers are provided to understand when the failures occurred.

Group (*n* = 20) Total Failures (Percentage)	Non Restorable	Restorable	Cycles	Load (N)
NFC 3 (15%)		1	83,950	600 N
		1	123,021	1300 N
		1	134,925	1400 N
NFA 2 (10%)	1		84,433	700 N
		1	103,975	900 N
FC 2 (10%)	1		137,112	1400 N
		1	132,650	1400 N
FA 2 (10%)	1		132,211	1400 N
		1	132,112	1400 N

**Table 2:** Percentage distribution of fractures across respective groups, including fatal and repairable failures, categorized into cohesive fractures within the tooth or restoration, loss of retention, and combined failures.

Group (*n* = 20)	Total Failures	Non-Restorable	Restorable	Cohesive Failure (Tooth)	Cohesive Failure (Restauration)	Combined Failure	Loss of Retention
NFC	3 (15%)		3	3	/		/
NFA	2 (10%)	1	1	2	/		/
FC	2 (10%)	1	1	1	/	1	/
FA	2 (10%)	1	1	1	/	1	/

**Table 3:** Comparison of survival times between the respective groups as calculated by the log-rank test. Significant *p*-values (*p* < 0.05) indicate a statistically significant difference.

Log-Rank Test	NFA	FC
NFC	*p* = 0.6551	*p* = 0.6209
FA	*p* = 0.9580	*p =* 0.9580

## Data Availability

The data presented in this study are available on request from the corresponding author.

## Abbreviations

Al_2_O_3_	Aluminum oxide
CEJ	Cemento-enamel junction
CEREC	Ceramic Reconstruction
FA	Ferrule, adhesive luting
FC	Ferrule, conventional cementation
GIC	Glass-ionomer cement
NaCl	Sodium chloride
NaN_3_	Sodium azide
NFA	No ferrule, adhesive luting
NFC	No ferrule, conventional cementation
